# Assessing curvilinear sprint performance in basketball: The role of stretch-shortening cycle and isometric force-time metrics

**DOI:** 10.1371/journal.pone.0356775

**Published:** 2026-08-26

**Authors:** Caner Mavili, Ekrem Yilmaz, Huseyin Celik, Evrim Unver, Sukru Alpan Cinemre

**Affiliations:** 1 Department of Exercise and Sport Sciences, Faculty of Sport Sciences, Hacettepe University, Ankara, Türkiye; 2 Department of Coaching Education, Faculty of Sport Sciences, Kirsehir Ahi Evran University, Kırşehir, Türkiye; 3 Department of Biomechanics and Motor Control, Faculty of Sport Sciences, Hacettepe University, Ankara, Türkiye; İzmir Democracy University: Izmir Demokrasi Universitesi, TÜRKIYE

## Abstract

Recent research emphasizes the importance of multidirectional movements in basketball. This cross-sectional study examines the differences in stretch-shortening cycle function, strength qualities, and dynamic and isometric force-time characteristics between basketball players with faster and slower curvilinear sprint (CS) performance. Forty-five competitive young male basketball players performed a basketball-specific three-point CS test and were divided into fast (*n* = 22) and slow (*n* = 23) groups based on their best CS times. Drop jump (DJ), countermovement jump (CMJ), and isometric mid-thigh pull (IMTP) tests were used to calculate the various force-time curve metrics. Independent samples Welch’s *t*-tests with Hedges’ *g* effect sizes and 95% confidence intervals were used to compare the force-time metrics between the two groups. In the DJ test, significant between-group differences (*p* < 0.05) with moderate to large effect sizes were observed in ground contact time (*g*=−0.896, 95% CI [−93.23, −19.28] ms) and its subphases (braking phase time and propulsion phase time), reactive strength index (*g* = 0.899, 95% CI [0.10, 0.49] m/s), and five other force-time metrics such as take-off peak force in body weight (*g* = 0.849, 95% CI [0.24, 1.33] BW). In the CMJ test, significant differences were mostly observed in braking-related metrics, including mean braking power (*g* = 0.789, 95% CI [0.52, 3.76] W/kg) and mean braking force (*g* = 0.732, 95% CI [0.03, 0.32] BW). In the IMTP test, no significant between-group differences were found in peak force (*p* = 0.224, *g* = 0.392), whereas all rate of force development (RFD) metrics showed significant differences with large to very large effect sizes (*g* = 0.822–1.407). The largest effect was observed for RFD at 150 ms (*g* = 1.407, 95% CI [1258.68, 3427.52] N/s), which closely matches previously reported ground contact times during CS, highlighting the importance of rapid force production within the brief contact phase that represents the sole window for actively modifying body momentum during locomotion. These findings suggest that DJ reactive strength index, CMJ braking force-time characteristics, and the rate of reaching maximal isometric force, rather than maximal isometric strength itself, in the IMTP test distinguish CS performance in male junior basketball players.

## Introduction

Basketball is one of the most popular team sports and is dominated by movements that produce high intensity in a short time (e.g., penetration, jumping, and sprinting; [1]. Most research has focused primarily on the linear aspects of such locomotion patterns, but recent research has increasingly focused on the multidirectional aspects of speed in sport [2]. This multidirectional approach has the potential to provide a more ecological context for field- and court-based sports because, for instance, the relatively small size of basketball courts and close contact with opponents make it difficult to perform many movements linearly [3]. In sport science, many locomotor patterns could be studied using this multidirectional approach; some of these movements are instantaneous in nature (e.g., change of direction), while others involve translation of the body over a timed duration (e.g., sprinting) [2]. For example, the ability to change direction, which is instantaneous in nature, has been shown to be crucial for sport performance in several studies (e.g., [4]. On the other hand, timed-duration actions in a multidirectional context, such as curvilinear sprinting (CS), have been investigated in only a few studies (e.g., [5–7].

While CS or bend sprinting, defined as upright sprint running in the presence of some degree of curvature [8], has been studied historically in track and field (e.g., [9], studies of CS in field- and court-based team sports have only recently emerged (e.g., [5,6]. In many attacking actions in team sports, to gain an advantage over opponents, the attacker attempts to evade the defender with curvilinear movements, usually sprinting, such as predator-prey analogy scenarios. Prey can escape from predators if the prey is faster than the predator, yet if they are in the same speed, the prey can only escape by CS and changing direction [10]. Despite this ecological context, in many team sports, linear sprint tests are often used alone among the various test content used to measure performance, and linear movements comprise almost all test procedures [5].

While linear movements constitute the major component of performance testing in most published studies on basketball (e.g., [11,12], and remain integral to athletic performance, the demands of the game are more multidirectional. Specifically, the speed demands of linear sprints, changes of direction, and CS constitute 48.3%, 20.7%, and 31.0% of total speed activity, respectively [13,14]. Therefore, the study of multidirectional movements, which are highly prevalent in the game, has the potential to be critical for understanding the performance demands and speed determinants of basketball. In particular, CS, which is performed to evade opponents both with the ball (e.g., penetrate) and without the ball (e.g., flare screen and cut, side-to-side off-ball movement, off-ball spacing) and involves curvilinear movement, has rarely been evaluated in previous studies (e.g., [5], although it is quite common in basketball.

To test CS performance on the field and court, [6] and Baena-Raya et al. [5] proposed new CS tests for soccer and basketball players, respectively, and examined the relationship between CS and linear sprint performance. In those studies, the authors suggested that, biomechanically, the two types of sprint running differ substantially in task execution and that linear sprinting and CS require different physical and technical skills. Although CS studies in team sports are relatively recent, research on track and field has shown that the force expression requirements of CS compared with linear sprinting differ (i.e., the need to generate centripetal force to negotiate the curvature). This leads to differences in force production biomechanics (e.g., peak vertical force) and running posture (e.g., inward lean) [9,15,16]. Additionally, in CS, the inward leg experiences greater horizontal braking impulses compared with the outward leg. Because the inward leg has less room to act due to the inward lean, it experiences longer braking phases [9,15,16]. In another study on curvilinear running at typical submaximal soccer velocities, Smith et al. [17] investigated foot-ground contact times (GCTs) at different radii and reported greater rearfoot and proportional ground contact times on the outward leg at tighter radii. These findings suggest that CS biomechanics differ from those of linear sprinting and may also differ among various track-, field-, and court-based sports, as the characteristics for locomotion (e.g., radii, distance, and context) may vary across sports. This implies that a sport-specific analysis of CS may be useful to better understand CS biomechanics in different sports and, specifically, in basketball for the present study.

The natural variation of muscle-tendon unit (MTU) function during locomotion often involves a stretch-shortening cycle (SSC), which may provide a useful theoretical framework for understanding the biomechanics underlying CS [18]. SSC is present in most movement patterns, such as sprinting and jumping, and involves a sequence of eccentric (lengthening) and concentric (shortening) contractions immediately afterward [18]. The specific biomechanics of any SSC function are influenced by the demands of the task, with Schmidtbleicher [19] suggesting that SSC can be categorized as either slow SSC (GCT > 250 ms) or fast SSC (GCT < 250 ms). Therefore, a detailed force-time analysis would allow us to examine the eccentric (braking) and concentric (propulsion) phases and type (i.e., fast or slow) of SSC and to closely analyze the force mechanics [20]. Reactive strength and enhanced expression of braking force have been shown to be kinetic determinants of linear sprint performance in highly trained track and field athletes (e.g., [21], whereas for sprint performance in basketball, few studies have performed a detailed force-time analysis. In other team sports, Loturco et al. (2020), for instance, examined the relationship between vertical jump (squat and countermovement) performance and both CS and linear sprint performance in young soccer players, finding varying relationships. Similarly, in youth basketball, Čaušević et al. [22] reported that countermovement jump (CMJ) and drop jump (DJ) performance were significantly correlated with linear sprint and agility performance across all age groups (U14, U15, and U16), with both jump measures emerging as the most significant predictors for sprint and agility variables, respectively. However, most studies, including Loturco et al. [23] and Čaušević et al. [22], examined jump height alone, which, while a fundamental performance indicator, may not be sufficient for a detailed biomechanical analysis [24]. Therefore, an analysis based on the phase and type of SSC could be critical to investigating CS performance with a more detailed understanding. To do so, we borrowed the approach of Cronin and Hansen [25], in which the authors classified SSC performance of rugby league players into slow and fast SSC, using the CMJ as a test of slow SSC and the DJ as a test of fast SSC. As CS studies in basketball are limited, understanding SSC function through a detailed force-time analysis in relation to CS performance has the potential to add new information to the literature.

In addition to CMJ and DJ tests, the isometric mid-thigh pull (IMTP) test [26] is another commonly used test that provides valuable insight into the strength qualities of athletes [27]. The IMTP test allows strength and conditioning practitioners and scientists to assess an athlete’s maximal isometric and explosive strength as well as their ability to produce force rapidly (i.e., rate of force development (RFD)) [27]. IMTP force-time metrics have been shown to correlate with performance in sprinting, jumping, and other power-demanding activities [28,29]. However, few studies have examined CS performance using the force-time metrics of the IMTP test.

Given the limitations in the current literature, the primary aim of this study was to examine differences in SSC function, strength qualities, and dynamic and isometric force-time characteristics, specifically DJ, CMJ, and IMTP force-time metrics, between basketball players with faster and slower CS performance. The independent variable was group classification (fast vs. slow), determined by a median split of the best CS times across four trials, while the dependent variables were the force-time metrics derived from the DJ, CMJ, and IMTP tests. Independent samples Welch’s t-tests with Hedges’ g effect sizes and 95% confidence intervals were used to evaluate between-group differences. We hypothesized that basketball players classified as fast in the CS test would exhibit significantly superior SSC function, strength qualities, and dynamic and isometric force-time characteristics than those classified as slow, with varying effect sizes depending on the metric examined.

## Methods

### Experimental approach

We used a cross-sectional design with force-time metrics to examine differences in SSC function, strength qualities, and dynamic and isometric force-time characteristics between athletes grouped as slow or fast based on a median split of CS times. All participants performed a validated and reliable basketball-specific CS test [5]. To assess SSC function, participants performed a DJ test from a 30 cm drop box as a measure of fast SSC and a CMJ test as a measure of slow SSC [25]. In addition, participants performed the IMTP test to assess strength qualities as well as the rate of force development. All force-time metrics from the jump and IMTP tests were obtained using a force plate (Kistler force plate, model 9260AA6, Switzerland)). The force-time curves were recorded using the MARS interface of the force plate, and each data file was then exported to MATLAB R2022b for analysis with custom-written code to calculate force-time metrics. Independent samples t-tests with Cohen’s *d* effect sizes were used to evaluate differences in SSC function, strength qualities, and force-time characteristics between the slow and fast groups. All participants underwent a familiarization period for each test one week prior to the beginning of the measurements. Measurements were conducted over three days, with a 48-hour rest period between sessions to minimize the effects of fatigue. On the first day, the DJ and CMJ tests were performed; on the second day, the CS and linear sprint tests were performed; and on the third day, the IMTP test was performed. Two successful trials were recorded for each measurement, and the best trial was used for analysis.

### Subjects

Forty-five young male basketball players (fast CS group: height: 190.27 ± 6.92 cm, weight: 81.71 ± 9.39 kg, age: 16.55 ± 0.51 years; slow CS group: height: 191.63 ± 8.01 cm, weight: 83.21 ± 12.50 kg, age: 16.26 ± 0.62 years) with no neuromusculoskeletal problems that would affect their test performance at the time of the measurements volunteered to participate in the study. To be eligible for the study and to minimize potential confounding factors, participants were required to meet all of the following criteria: (1) be a young male basketball player aged 15–18 years, (2) be actively competing in official basketball competitions, (3) have been playing basketball at a competitive level for at least four years, (4) have a weekly training volume of at least four days and eight hours, and (5) be classified as post-peak height velocity (post-PHV) according to the Mirwald equation. Participants were excluded if they (1) did not meet the post-PHV criterion, or (2) had sustained a major lower-extremity injury (e.g., Achilles tendon rupture or ACL tear) within the previous year. No injuries occurred during the study period. Prior to testing, participants were not subjected to any intervention outside their regular training routine and did not participate in any competition within 72 hours of testing. All participants and their families provided written informed consent prior to participation. Participants were enrolled between 20 February 2024 and 4 February 2025, during which time data were collected. The study procedures were approved by the Hacettepe University Health Sciences and Research Ethics Committee [SBA24/064].

For sample size estimation, using G*Power (v.3.1) (Faul et al., 2007), we performed an a priori statistical power analysis of the sample size required for a two-tailed t-test to compare differences between two independent means. Based on the effect size of 0.94, converted from the pooled correlation (r = −0.426) between reactive strength index and sprint performance reported in the meta-analysis by Jarvis et al. [30], a minimum total sample size of 38 participants (19 per group) was calculated for alpha = 0.05 and power = 0.80. We recruited 45 young male basketball players from the youth teams of professional basketball clubs that competed at the highest division in their respective junior basketball state leagues and had reached the final four of their competitive leagues at the end of the season.

### Procedures

#### Anthropometrics.

All anthropometric measurements were conducted on the same day. Participants’ height was assessed using a portable stadiometer (Holtain, United Kingdom), while body weight was measured with a scale (TANITA, Japan). Sitting height was measured using a stadiometer with the knees flexed at 90°, and torso length was calculated by subtracting sitting height from standing height [31]. Biological maturation status was also assessed to ensure that all participants were in the post-PHV group according to the Mirwald equation [32]. Anthropometric measurements are presented in Table 1.

**Table 1:** Height, weight, and age measures of participants.

	Fast CS Group		Slow CS Group						
	Mean	SD	Mean	SD	Mean diff.	*p*	Hedges’ *g*	CI Lower	CI Upper
Height (cm)	190.273	6.923	191.626	8.006	−1.353	0.547	−0.178	−5.848	3.142
Weight (kg)	81.714	9.388	83.213	12.504	−1.499	0.651	−0.133	−8.139	5.140
Age (years)	16.545	0.510	16.261	0.619	0.285	0.099	0.493	−0.056	0.625

Age = chronological age, SD = standard deviation, CI = 95% confidence interval

#### Drop jump test.

A DJ test was performed in an akimbo pose from a 30 cm height onto the force plate with a sampling frequency of 1000 Hz. The DJ technique was explained in detail to the participants during both familiarization and test sessions. Participants were verbally instructed to minimize ground contact time and jump as high as possible, with the force plate conceptualized as a hot plate. The rest period between jumps was approximately one minute. In the force-time analysis, the DJ contact period was divided into two phases: eccentric (braking or landing) and concentric (propulsion or take-off) [33]. For detailed examination of these phases, a total of 16 metrics were analyzed, including average and peak force, average and peak power, time in each phase, impulse of each phase, and spring-like correlation. The names, calculations, and units of these force-time metrics are listed in Appendix A. Specifically, the spring-like correlation (SLC) is part of a relatively new method proposed by Pedley and colleagues [20] to categorize stretch-shortening cycle (SSC) performance based on the Pearson correlation between vertical ground reaction force (VGRF) and body center of mass (BCOM) displacement during foot-ground contact. Subjects’ spring-like behavior (SLB) was categorized according to the presence of an impact peak (defined as the highest visible transient peak during the braking phase, occurring in the first 20% of foot-ground contact) and their SLC score. These categories were: good (no impact peak and SLC <−0.80), moderate (impact peak present but SLC <−0.80), and poor (impact peak present and SLC ≥ −0.80). Note that the negative sign in the inequality indicates that the absolute value of the SLC lies between 0.80 and 1.00 (perfect linearity) for acceptable SLB.

#### Countermovement jump test.

During the CMJ trials, participants placed their hands on their waists and were instructed to drop into a self-selected countermovement depth and then jump as high as possible immediately after the countermovement phase, landing back on the force plate. Each participant performed two trials with approximately one minute of rest between jumps, and the force-time metrics obtained from the highest jump were used for analysis. The CMJ test force-time curve was divided into several phases, including eccentric (braking) and concentric (propulsion), to examine SSC function in detail. A total of 36 metrics, including reactive strength index modified (RSImod), jump height (JH), and time to take-off (TTT), were used for analysis [34,35]. The names, calculations, and units of the CMJ metrics are listed in Appendix B. The force-time metrics obtained from the CMJ test are widely used in basketball research and have exhibited adequate intra- and inter-session reliability [36,37].

#### Isometric mid-thigh pull test.

In the IMTP test, the athlete assumes an upright position with knees extended at approximately 120–140 degrees and both feet placed on the force platform in the isometric “second pull” position. The test is performed by pulling a fixed bar with maximal effort using both hands [26,38]. As a result of the test, maximal isometric force, impulse, and RFD can be calculated with high accuracy. The IMTP is considered one of the safest tests for strength assessment in athletes [26]. During the tests, the trunk remains upright, the feet are positioned under the bar and centered on the force platform, and they are aligned with the participant’s foot span, approximately centered on the bar. The knees remain in light contact with the bar. Participants were instructed to pull as hard and as fast as possible while pushing their feet into the ground [39]. The IMTP test was assessed using a total of 17 metrics, including maximal isometric strength, peak RFD during a 20-millisecond window (pRFD20) [40], as well as force, RFD, and impulse in five different time windows [41]. In the analysis of IMTP force-time curve, the manual force onset identification method evaluated by Guppy et al. [42] was used. This method has been shown to have excellent intra-rater reliability (ICC = 1.00 [0.99, 1.00,^42^]. The force onset was identified by a single experienced rater (the first author) using the *ginput* function within MATLAB. The determination of the force onset was based on the method described by [43] and [44]), which identifies the last peak or trough before the signal deflects away from baseline noise. The names, calculations, and units of the IMTP force-time metrics are listed in Appendix C.

#### Curvilinear sprint test.

A novel CS test developed by Baena-Raya et al. [5] was used to assess athletes’ sprint performance. The test was performed around the 3-point line on a basketball court, covering a total distance of 18.7 m. We also recorded 5 m split times during the CS test to assess initial acceleration. During the test, the subjects’ front foot was placed 0.5 m before the first timing gate in a split-stance start position. The test began at the participants’ discretion once the timing gate system was ready. Times were recorded using photocells (Fusion Smart Speed, Australia). Timing gates were positioned at a height of 0.75 m and a width of 1.5 m. Measurements were performed on both the right and left sides of the three-point line at maximal effort, with at least a 3-minute rest period between trials. Before testing, subjects were instructed to run as quickly as possible. This three-point line CS test has been shown to have high relative and absolute reliability for CS sprint time on both the right side (ICC = 0.93; CV = 1.65%) and the left side (ICC = 0.94; CV = 1.54%) in young basketball players [5]. The CS test was performed on both sides of the three-point line, with two trials per side; the best performance, based on completion time at 18.7 m across the four trials, was selected for further analysis. The median split method was then used to classify participants into two groups based on their best CS time: fast (*n* = 22) and slow (*n* = 23).

#### Linear sprint test.

The linear sprint performance of the athletes was measured using photocells on a 18.7 m straight run. The photocells, which were placed at 5 m and 18.7 m, recorded the times to assess 5 m acceleration and 18.7 m linear sprint performance [5].

### Statistical analysis

Descriptive statistics for the fast and slow groups were reported as the mean and the standard deviation. This study employed a cross-sectional comparative design with two independent groups (fast and slow CS groups). The dependent variables comprised the force-time metrics derived from the DJ, CMJ, and IMTP tests. Prior to conducting the comparisons, the normality of all variables was assessed and verified using the Shapiro-Wilk test and Q-Q plots. To test the null hypothesis of equal population means against the alternative hypothesis of unequal means between the fast and slow groups, an independent samples Welch’s *t*-test was used instead of Student’s *t*-test. This selection was based on the recommendation of Delacre, Lakens, and Leys [45], who advise using Welch’s t-test by default, as it performs better when sample sizes and variances are unequal between groups and yields equivalent results when they are equal, thereby eliminating the need for a preliminary Levene’s test, which often lacks the power to detect unequal variances. The statistical significance was taken as p < 0.05.

All statistical analyses (except sample size and sensitivity estimates) were performed in R (version 4.5.0; R Core [46] using Welch’s independent samples *t*-test to compare fast and slow groups, with effect sizes reported as Hedges’ *g* (denoted as *g*) computed from the non-pooled standard deviation with bias correction as described by Delacre et al. [47]. The effect size classification used in this study was adapted from Cohen [48] and Hopkins [49]: minimal (*g* < 0.20), small (*g* = 0.20–0.50), moderate (*g* = 0.50–0.80), large (*g* = 0.80–1.20), and very large (*g* > 1.20).

A sensitivity analysis was conducted using G*Power software (version 3.1; Faul et al., 2007) to determine the minimum detectable effect size given our sample size, an alpha level of 0.05, and a statistical power of 0.80 for a two-tailed independent samples t-test. The minimum detectable effect size was 0.855, indicating that the study was adequately powered to detect large effects.

## Results

The height, weight, and age of the participants are presented in Table 1. When the mean values of the groups were analyzed, no significant differences were observed for height, weight, and age (p > 0.05). Additionally, all participants were found to be in post-PHV according to the Mirwald equation [32].

Table 2 shows the scores of the fast and slow CS groups and the sprint performance differences between the two groups. A significant difference was observed between the fast and slow groups in the 5 m linear sprint time, while no significant difference was observed in the 18.7 m linear sprint time. When the results of the CS test of the participants were analyzed, significant differences were observed in both 5 m CS time and 18.7 m CS time of the fast CS group compared to the slow CS group.

**Table 2:** Curvilinear and linear sprint test results of participants.

	Fast CS Group		Slow CS Group						
	Mean	SD	Mean	SD	Mean diff.	*p*	Hedges’ *g*	CI Lower	CI Upper
LS 5 m (s)	0.908	0.071	0.993	0.061	−0.085	**<0.001**	**−1.263**	−0.125	−0.045
LS 18.7 m (s)	2.762	0.093	2.799	0.131	−0.036	0.286	−0.315	−0.105	0.032
CS 5 m (s)	0.872	0.092	1.020	0.092	−0.148	**<0.001**	**−1.578**	−0.204	−0.093
CS 18.7 m (s)	2.925	0.098	3.294	0.143	−0.369	**<0.001**	**−2.961**	−0.443	−0.296

LS = linear sprint, CS = curvilinear sprint, SD = standard deviation, CI = 95% confidence interval

Table 3 shows the results of the DJ test comparisons between the fast and slow CS groups. According to this table, the fast CS group obtained significantly better values than the slow CS group in the following metrics: ground contact time (GCT) (p = 0.004, g = 0.896, large effect), reactive strength index (RSI) (p = 0.004, g = 0.899, large effect), average power concentric phase (TPow) (p = 0.009, g = 0.807, large effect), peak vertical ground reaction force (pVGRF) (p = 0.022, g = 0.696, moderate effect), landing peak force (LPF) (p = 0.029, g = 0.666, moderate effect), and take off peak force (TPF) (p = 0.006, g = 0.849, large effect). In addition, the ground contact time for the fast CS group was significantly shorter in both the braking phase time (BPT) (p = 0.006, g = 0.854, large effect) and propulsion phase time (PPT) (p = 0.013, g = 0.758, moderate effect) phases. In addition, the fast CS group had significantly higher values for the normalized vertical stiffness (KvertNorm) metric (p = 0.014, g = 0.763, moderate effect) compared to the slow CS group. Although there was no significant difference in the SLC metric (p > 0.05), according to the spring like behavior results, 16 out of 22 participants in the fast CS group were in the good category (72.73%) and 6 of them were in the moderate category (27.27%). However, in the slow CS group, although 14 out of 23 participants (60.87%) were in the good category, 4 participants were in the moderate (17.39%) category, and 5 participants were in the poor (21.74%) category.

**Table 3:** Descriptive statistics and comparative analyses for the DJ test metrics.

	Fast CS Group		Slow CS Group						
Metric	Mean	SD	Mean	SD	Mean diff.	*p*	Hedges’ *g*	CI Lower	CI Upper
JH	31.074	4.611	29.566	4.685	1.507	0.283	0.319	-1.288	4.303
GCT	239.136	39.516	295.391	77.243	-56.255	**0.004**	**-0.896**	-93.232	-19.278
RSI	1.347	0.364	1.053	0.270	0.294	**0.004**	**0.899**	0.100	0.488
LPow	33.711	10.414	30.018	6.925	3.693	0.172	0.409	-1.677	9.063
TPow	42.717	8.365	36.617	6.327	6.099	**0.009**	**0.807**	1.612	10.586
PCOMD	14.493	6.501	17.265	4.295	-2.772	0.102	-0.493	-6.118	0.574
pVGRF	5.023	0.960	4.388	0.827	0.635	**0.022**	**0.696**	0.094	1.175
LPF	4.942	1.020	4.306	0.846	0.636	**0.029**	**0.666**	0.070	1.201
TPF	4.721	0.927	3.937	0.885	0.783	**0.006**	**0.849**	0.238	1.329
LTTD	16.514	12.513	25.241	16.681	-8.727	0.053	-0.581	-17.582	0.127
KvertNorm	451.390	174.870	340.993	97.163	110.397	**0.014**	**0.763**	24.021	196.772
BPT	101.773	25.115	128.783	35.952	-27.010	**0.006**	**-0.854**	-45.637	-8.382
PPT	137.364	22.782	166.609	48.089	-29.245	**0.013**	**-0.758**	-51.948	-6.542
Braking Impulse	0.299	0.065	0.332	0.045	-0.032	0.060	-0.569	-0.066	0.001
Propulsion Impulse	0.400	0.039	0.422	0.064	-0.022	0.175	-0.402	-0.054	0.010
SLC	-0.865	0.103	-0.859	0.112	-0.006	0.851	0.056	-0.059	0.071

JH = jump height (cm), GCT = ground contact time (ms), RSI = reactive strength index (m/s), LPow = average power eccentric phase (W/kg), TPow = average power concentric phase (W/kg), PCOMD = peak center of mass displacement (cm), pVGRF = peak vertical ground reaction force (BW), LPF = landing peak force (BW), TPF = take-off peak force (BW), LTTD = landing to take-off time difference (%), KvertNorm = normalized vertical stiffness (N/m/kg), BPT = braking phase time (ms), PPT = propulsion phase time (ms), Braking Impulse = braking impulse (BW·s), Propulsion Impulse = propulsion impulse (BW·s), SLC = spring-like correlation, BW = body weight (N), SD = standard deviation, CI = 95% confidence interval

Table 4 shows the comparison of the CMJ jump test between the two groups. According to this table, significant differences were observed between the fast CS group and the slow CS group in the braking phase metrics. When examining the metrics, the fast CS group showed significantly better performance compared to the slow CS group in the following: braking phase time (BPT) (p = 0.044, g = 0.616, moderate effect), mean braking force (MBF) (p = 0.018, g = 0.732, moderate effect), mean braking power (MBP) (p = 0.011, g = 0.789, moderate effect), mean braking velocity (MBV) (p = 0.015, g = 0.752, moderate effect), mean propulsion velocity (MPV) (p = 0.015, g = 0.748, moderate effect), peak braking force (PBF) (p = 0.025, g = 0.691, moderate effect), peak braking power (PBP) (p = 0.038, g = 0.636, moderate effect), and net braking impulse (p = 0.049, g = 0.602, moderate effect). No significant differences were observed between the two groups in any of the remaining metrics.

**Table 4:** Descriptive statistics and comparative analyses for the CMJ test metrics.

	Fast CS Group		Slow CS Group						
Metric	Mean	SD	Mean	SD	Mean diff.	*p*	Hedges’ *g*	CI Lower	CI Upper
TTT	0.677	0.085	0.709	0.089	-0.031	0.244	-0.350	-0.084	0.022
FT	0.545	0.038	0.530	0.032	0.016	0.150	0.435	-0.006	0.037
JH	36.594	5.137	34.508	4.215	2.086	0.149	0.436	-0.008	0.049
RSI	0.817	0.118	0.760	0.113	0.057	0.106	0.489	-0.013	0.128
mRSI	0.549	0.106	0.494	0.086	0.055	0.068	0.555	-0.004	0.114
UPT	0.317	0.043	0.322	0.050	-0.006	0.692	-0.118	-0.034	0.023
BPT	0.134	0.026	0.151	0.026	-0.016	**0.044**	**-0.616**	-0.032	0.000
PPT	0.227	0.032	0.236	0.032	-0.009	0.358	-0.275	-0.028	0.011
MBF	1.951	0.232	1.774	0.244	0.178	**0.018**	**0.732**	0.033	0.323
MPF	2.203	0.195	2.127	0.176	0.076	0.185	0.399	-0.037	0.189
MBP	13.219	2.598	11.081	2.719	2.138	**0.011**	**0.789**	0.519	3.756
MPP	32.103	4.085	29.960	3.473	2.143	0.068	0.555	-0.165	4.452
MBV	0.775	0.096	0.701	0.098	0.075	**0.015**	**0.752**	0.015	0.134
MPV	1.682	0.104	1.604	0.103	0.079	**0.015**	**0.748**	0.016	0.142
PBF	2.662	0.392	2.361	0.459	0.300	**0.025**	**0.691**	0.041	0.560
PPF	2.752	0.363	2.648	0.408	0.104	0.377	0.264	-0.131	0.339
PBP	17.890	3.809	15.235	4.367	2.654	**0.038**	**0.636**	0.160	5.149
PPP	55.425	6.650	53.656	5.335	1.769	0.336	0.288	-1.904	5.443
PBV	1.195	0.159	1.090	0.186	0.105	0.050	0.597	0.000	0.211
PPV	2.777	0.174	2.715	0.144	0.062	0.202	0.384	-0.035	0.160
NBI	1.203	0.159	1.097	0.187	0.106	**0.049**	**0.602**	0.001	0.212
NPI	2.630	0.183	2.565	0.152	0.066	0.203	0.383	-0.037	0.168
NIR	2.227	0.366	2.402	0.429	-0.175	0.153	-0.431	-0.418	0.068
BPW	57.891	13.887	50.551	20.048	7.341	0.166	0.417	-3.191	17.872
PPW	279.543	42.024	268.621	42.459	10.922	0.396	0.254	-14.781	36.625
NWR	5.187	2.020	6.044	2.262	-0.857	0.192	-0.392	-2.163	0.448
CMD	0.252	0.036	0.260	0.053	-0.008	0.558	-0.175	-0.036	0.019
FMD	2.654	0.401	2.353	0.467	0.302	**0.027**	**0.680**	0.037	0.567
JM	213.255	24.491	210.666	30.832	2.589	0.759	0.091	-14.378	19.556
BPR	19.741	2.140	21.186	2.078	-1.445	**0.028**	**-0.673**	-2.729	-0.162
PPR	33.448	2.333	33.326	2.869	0.122	0.878	0.046	-1.471	1.715
UPR	46.811	3.773	45.487	4.019	1.323	0.267	0.333	-1.049	3.695
AST	8.655	2.571	7.715	3.356	0.940	0.303	0.308	-0.883	2.763
NST	106.970	29.845	94.899	39.819	12.071	0.262	0.336	-9.389	33.532
URFD	3.717	1.221	3.446	1.509	0.272	0.515	0.194	-0.565	1.108
ERFD	10.314	4.325	7.717	4.222	2.597	0.050	0.597	-0.004	5.198

TTT = time-to-take-off (s), FT = flight time (s), JH = jump height (cm), RSI = reactive strength index (s/s), mRSI = modified reactive strength index (m/s), UPT = unweighting phase time (s), BPT = braking phase time (s), PPT = propulsive phase time (s), MBF = mean braking force (BW), MPF = mean propulsion force (BW), MBP = mean braking power (W/kg), MPP = mean propulsion power (W/kg), MBV = mean braking velocity (m/s), MPV = mean propulsion velocity (m/s), PBF = peak braking force (BW), PPF = peak propulsion force (BW), PBP = peak braking power (W/kg), PPP = peak propulsion power (W/kg), PBV = peak braking velocity (m/s), PPV = peak propulsion velocity (m/s), NBI = net braking impulse (m/s), NPI = net propulsion impulse (m/s), NIR = net impulse ratio, BPW = braking phase work (J), PPW = propulsion phase work (J), NWR = net work ratio, CMD = countermovement depth (m), FMD = force at minimum displacement (BW), JM = jump momentum (kg·m/s), BPR = braking phase ratio (%), PPR = propulsion phase ratio (%), UPR = unweighting phase ratio (%), AST = absolute stiffness (kN/m), NST = normalized stiffness (N/m/kg), URFD = unweighting phase rate of force development (BW/s), ERFD = eccentric rate of force development (BW/s), BW = body weight (N), SD = standard deviation, CI = 95% confidence interval

Table 5 shows the comparison of IMTP test metrics between the fast CS group and the slow CS group. According to the IMTP test results, there was no significant difference between the two groups in the force metrics except at 150 ms (p = 0.030, g = 0.713, moderate effect). Similarly, there were no significant differences in impulse values. However, when examining the RFD values, the fast CS group achieved significantly higher values compared to the slow CS group in the following metrics: peak rate of force development (Max pRFD20) (p = 0.002, g = 1.140, large effect), RFD 50 ms (p = 0.008, g = 0.934, large effect), RFD 90 ms (p = 0.001, g = 1.205, very large effect), RFD 150 ms (p < 0.001, g = 1.407, very large effect), RFD 200 ms (p = 0.002, g = 1.046, large effect), and RFD 250 ms (p = 0.014, g = 0.822, large effect).

**Table 5:** Descriptive statistics and comparative analyses for the IMTP test metrics.

	Fast CS Group		Slow CS Group						
Metric	Mean	SD	Mean	SD	Mean diff.	*p*	Hedges’ *g*	CI Lower	CI Upper
Peak Force	2366.571	306.936	2232.692	359.271	133.879	0.224	0.392	−85.395	353.153
Force 50 ms	1087.074	183.924	1069.470	388.328	17.605	0.857	0.056	−181.287	216.497
Force 90 ms	1348.819	270.444	1188.744	378.511	160.075	0.140	0.476	−55.180	375.330
Force 150 ms	1636.094	267.983	1397.664	376.322	238.431	**0.030**	**0.713**	24.668	452.193
Force 200 ms	1814.622	283.926	1620.415	401.421	194.206	0.092	0.546	−33.282	421.695
Force 250 ms	1937.108	277.591	1791.264	402.226	145.844	0.199	0.412	−80.225	371.913
Max pRFD20	10423.317	6046.611	5012.071	2330.268	5411.246	**0.002**	**1.140**	2260.007	8562.485
RFD 50 ms	3977.584	3631.621	1364.809	1149.903	2612.775	**0.008**	**0.934**	748.770	4476.780
RFD 90 ms	5118.045	3119.770	2083.501	1470.885	3034.544	**0.001**	**1.205**	1370.635	4698.454
RFD 150 ms	4985.996	1829.560	2642.899	1394.559	2343.097	**<0.001**	**1.407**	1258.678	3427.516
RFD 200 ms	4632.133	1424.778	3095.930	1449.838	1536.202	**0.002**	**1.046**	589.267	2483.138
RFD 250 ms	4195.653	1142.464	3160.141	1317.929	1035.513	**0.014**	**0.822**	225.867	1845.158
IMP 50 ms	48.146	5.878	51.290	19.224	−3.144	0.493	−0.214	−12.489	6.201
IMP 90 ms	96.940	14.094	96.411	34.432	0.530	0.950	0.020	−16.715	17.775
IMP 150 ms	186.979	29.310	173.787	56.233	13.192	0.365	0.286	−16.140	42.525
IMP 200 ms	273.427	41.025	249.120	74.050	24.307	0.215	0.396	−14.840	63.454
IMP 250 ms	367.472	52.578	334.934	92.262	32.538	0.186	0.422	−16.564	81.640

Peak Force = peak force value on the force-time curve (N), Force 50 ms (millisecond) = force at 50 ms (N), Force 90 ms = force at 90 ms (N), Force 150 ms = force at 150 ms (N), Force 200 ms = force at 200 ms (N), Force 250 ms = force at 250 ms (N), Max pRFD20 = peak rate of force development during a 20-millisecond window (N/s), RFD 50 ms = rate of force fevelopment at 50 ms window (N/s), RFD 90 ms = rate of force development at 90 ms window (N/s), RFD 150 ms = rate of force development at 150 ms window (N/s), RFD 200 ms = rate of force development at 200 ms window (N/s), RFD 250 ms = rate of force development at 250 ms window (N/s), IMP 50 ms = impulse at 50 ms window (N·s), IMP 90 ms = impulse at 90 ms window (N·s), IMP 150 ms = impulse at 150 ms window (N·s), IMP 200 ms = impulse at 200 ms window (N·s), IMP 250 ms = impulse at 250 ms window (N·s), SD = standard deviation, CI = 95% confidence interval

## Discussion

In this study, we examined differences in SSC function, strength qualities, and dynamic and isometric force-time characteristics between basketball players with faster and slower CS performance. Based on the DJ metrics, significant between-group differences with moderate to large effect sizes were observed in GCT and its subphases (BPT and PPT), RSI, peak force metrics (LPF and TPF), average concentric power (TPow), normalized vertical stiffness, and pVGRF. The CMJ analysis revealed significant differences primarily in braking phase metrics (BPT, MBF, MBP, MBV, PBF, PBP, and net braking impulse), with moderate effect sizes. The IMTP results showed that the fast CS group produced significantly higher values than the slow CS group across all RFD metrics (Max pRFD20 and the 50, 90, 150, 200, and 250 ms windows), with effect sizes ranging from large to very large, as well as in force at 150 ms. Our hypotheses were partially supported, as significant differences between the fast and slow CS groups were observed for several force-time metrics, with varying effect sizes.

One of the main findings of the DJ test was that the fast and slow CS groups did not differ in JH, yet the fast group had significantly higher RSI values due to significantly shorter GCTs. Because RSI reflects the ability to rapidly transition from eccentric to concentric MTU action during a fast SSC task, this indicates greater reactive strength in the fast CS group. Similar results have been reported in linear sprinting, where reactive strength has been identified as a key determinant of sprint performance (e.g., [21,50]. Furthermore, a recent meta-analysis reported a significant moderate-to-large relationship between RSI and sprint and change-of-direction performance across athletes from various sports and competition levels, including basketball, soccer, and sprinting [30]. In line with reactive strength, the fast CS group also produced significantly higher peak forces in braking and propulsion phases (i.e., LPF and TPF). Given that JH, and therefore net vertical impulse (including its braking and propulsion subphases), was comparable between groups, the higher peak forces in the fast group reflect a different force-expression strategy: applying greater force to the ground over a shorter contact time. Douglas et al. [21] reported the same pattern in highly trained sprinters, where greater force application within shorter GCTs distinguished highly trained sprint athletes from non-sprint-trained, physically active participants. These findings suggest that reactive strength, expressed as the capacity to generate relatively higher forces within shorter GCTs, is a quality associated with CS performance in basketball.

The SLB classification proposed by Pedley et al. [20] provides a more detailed framework for assessing SSC function during a DJ test by combining the presence of an impact peak with the SLC. In our study, no significant between-group difference was observed in the SLC values themselves; however, the categorical SLB distribution differed between groups. The fast CS group comprised 72.73% good and 27.27% moderate SLB classifications, with no participants in the poor category, whereas the slow CS group comprised 60.87% good, 17.39% moderate, and 21.74% poor. This indicates that the between-group difference in SLB was driven primarily by the absence of an impact peak rather than by differences in SLC values. In their study on youth soccer players, Pedley et al. [20] reported that players classified as good exhibited significantly shorter GCTs, greater take-off peak forces, and higher RSI values than those classified as moderate or poor. In line with these findings, the fast CS group in our study showed shorter GCTs, higher RSI, and greater peak forces alongside a more favorable SLB distribution. To our knowledge, this is the first study to examine SLB in relation to CS performance in basketball. These preliminary findings tentatively suggest that effective spring-like behavior, expressed as the absence of an impact peak during landing, may be a relevant aspect of SSC function contributing to CS performance in youth basketball.

The CMJ test results indicated that significant between-group differences were largely confined to braking-phase metrics, with the fast CS group exhibiting greater mean and peak braking force, power, and velocity, as well as a shorter braking phase time, compared with the slow CS group. As the CMJ was used in this study as a measure of slow SSC function, these findings suggest that the slow SSC contribution to CS performance is expressed primarily through the eccentric (braking) phase rather than the concentric (propulsion) phase. This braking-specific pattern aligns with the biomechanical demands of CS, in which the requirement to generate centripetal force to follow the curved path results in shortened flight time and step length, increased ground contact time, and greater braking impulse compared with linear sprinting. In line with CMJ braking characteristics for sprint performance, Beattie et al. [51] compared world-class elite and sub-elite male sprinters and reported very large between-group differences in braking impulse and braking peak velocity, as well as a large difference in braking peak power, although braking peak force showed only a moderate, non-significant difference. Nishiumi et al. [52] further examined the force-velocity profile during the braking phase of the CMJ and reported a linear relationship between mean braking force and braking velocity. They found that while both the slope and the theoretical maximum braking force significantly correlated with multi-joint eccentric strength, only the relative theoretical maximum braking force significantly correlated with braking peak force and jump height. The authors also suggested that increased braking phase force may enhance force production in the early concentric phase, contributing to overall performance. Overall, our findings suggest that CMJ braking-phase characteristics, which reflect slow SSC function during the eccentric phase, may be a useful measure of lower-limb capacity to absorb and redirect forces, a demand that appears relevant to CS performance in youth basketball.

The IMTP results showed that the fast and slow CS groups differed across all RFD windows, whereas no meaningful between-group differences were observed in force or impulse metrics. As in the IMTP test, impulse (i.e., the total force applied over time [53]) related metrics showed no differences between the fast and slow CS groups, as net impulse values were similar in the DJ and CMJ tests. These findings indicate that it is not the overall magnitude of force application during IMTP, DJ, and CMJ tests, but rather the rapid force expression, that differentiates athletes across isometric, fast SSC, and slow SSC actions. RFD is a key measure derived from the IMTP test that reflects the rate at which the MTU generates force within a given time interval. Higher RFD values indicate an athlete’s ability to produce force rapidly, which is particularly advantageous in movements with relatively short ground contact times, such as sprinting and jumping [29]. Our RFD findings are consistent with previous reports of large to very large inverse relationships between IMTP RFD and linear sprint performance over 5–20 m in collegiate athletes and rugby union players [29,54,55]. In contrast to the RFD metrics, IMTP PF, which is a measure of maximal isometric strength, did not differ between the fast and slow CS groups. Several studies (e.g., [29] suggest that linear sprinting performance in basketball players generally improves as strength capabilities increase, yet recent research indicates that speed does not always scale proportionally with strength gains. For instance, Vial et al. [56] showed that sprint speed does not always scale linearly with maximal strength gains, possibly due to a saturation effect whereby further increases in certain strength qualities yield diminishing returns once sufficient force-generating capacity has been reached. In another study, Donaldson et al. [57] suggested that the relationship between strength expression and sprint performance may involve complex nonlinear interactions with coordination patterns. Therefore, maximal isometric strength on its own may not differentiate athletes when other neuromuscular and coordination qualities also contribute to sprint speed, and such qualities may be more pronounced for CS. In summary, our results suggest that faster CS performance is associated with higher RFD, which reflects the MTU’s ability to generate force rapidly, an attribute important for activities requiring high-speed force production, such as sprinting [58]. Higher RFD capacity could help athletes reach higher speeds in a shorter time, particularly during the sprint start and acceleration phases, and can therefore be considered a potential correlate of sprint performance [59].

To evaluate CS performance, this study analyzed SSC function using two distinct jump tests: the DJ as a test of fast SSC and the CMJ as a test of slow SSC [25]. In the DJ test, the fast CS group demonstrated shorter ground contact times (*g* = −0.896), higher reactive strength index (*g* = 0.899), and greater peak forces (*g* = 0.666–0.849), all with moderate to large effect sizes. From a practical standpoint, these large effects suggest that the ability to produce high forces rapidly during short ground contact time (i.e., reactive strength (e.g., [27]) has the potential to distinguish faster from slower CS performers. Therefore, basketball practitioners aiming to improve CS performance may benefit from incorporating plyometric exercises that emphasize fast SSC function, such as drop jumps, to develop reactive strength qualities (e.g., [60]. In the CMJ test, significant between-group differences were observed in braking-related metrics, with moderate effect sizes (e.g., mean braking power, *g* = 0.789; mean braking force, *g* = 0.732). These findings suggest that the capacity to absorb and redirect forces during the braking phase of slow SSC movements is associated with CS performance and serves as a general indicator of the lower-limb eccentric capacity required to manage the complex, multidirectional deceleration demands of maintaining the running axis on curvilinear paths. For practitioners, this indicates that training interventions targeting eccentric strength and braking force production during the countermovement phase may be relevant to CS development. In the IMTP test, no significant differences were observed in PF between the groups (*g* = 0.392, small effect), whereas all RFD metrics differed significantly with large to very large effect sizes (*g* = 0.822–1.407). Notably, the largest effect was observed for RFD at 150 ms (*g* = 1.407), which is considered a very large effect. These large to very large effects indicate that the rate at which force is generated, rather than the magnitude of maximal force itself, is the more distinguishing factor between faster and slower CS performers. Additionally, the 150 ms RFD window, which produced the largest effect size in IMTP metrics, matches very closely with the GCTs reported during CS by [6]: 148 ms for the outward leg and 137 ms for the inward leg. During terrestrial locomotion, the ground contact phase represents the sole temporal interval in which an animal can actively modify its linear and angular momentum, as it is the only period when ground reaction forces can generate a net impulse on the body [10]. In practical terms, these findings suggest that once a sufficient level of maximal strength is achieved, further improvements in CS performance may depend more on the ability to express force rapidly. Consequently, once foundational maximal strength is established, strength and conditioning frameworks for basketball players may benefit from prioritizing interventions designed to optimize rapid force expression. Taken together, our findings suggest that CS is a complex movement pattern in which both fast and slow SSC function, as well as the rate of force development, are associated with performance. From a training perspective, basketball practitioners should consider developing both reactive and braking force qualities alongside rapid force expression capabilities to support CS performance.

### Limitations

Our study has certain limitations. Although the three-point line CS test has been shown to be a highly reliable test for assessing CS performance in young basketball players [5], a sample-specific test-retest reliability analysis was not performed in the present study. Future studies should consider reporting reliability metrics within their own samples to further strengthen the validity of the CS test across different populations and testing environments.

Our study used a cross-sectional comparative design, and the nature of this study design does not allow for causal inferences. Although significant between-group differences were observed in several force-time metrics, these findings reflect associations rather than causal relationships. Further experimental studies are needed to investigate whether and to what extent targeted improvements in specific force-time metrics lead to improvements in CS performance.

The force onset in the IMTP test was determined by a single rater. Although the manual identification method has been shown to have excellent intra-rater reliability [42], inter-rater reliability was not assessed in the present study. Given the degree of subjectivity inherent to manual identification, there might be some variation in the identified instant of force onset between raters, with the accuracy of the method being at least partially dependent on the experience of the individual performing the analysis [42]. Future studies should consider assessing inter-rater reliability when using this method.

Additionally, in statistical analysis, we performed multiple independent samples t-tests on DJ, CMJ, and IMTP metrics without correction for multiple comparisons. The necessity and appropriateness of corrections for multiple comparisons is a debated topic in the statistical literature, with some researchers arguing against routine due to the potential of masking meaningful differences as well as arbitrary decisions on how many other tests were performed [61–63], while others recommend accounting for multiple testing to control for inflated Type I error rates [64]. In our data analysis, each test addressed a distinct biomechanical variable, and effect sizes were reported to facilitate the evaluation of practical significance [61–63]; however, the possibility of inflated Type I error rates should be considered when interpreting the results.

Another limitation is that our analysis was based on whole-body force-time curves obtained from the DJ, CMJ, and IMTP tests rather than on neuromuscular activity at the individual muscle or joint level. Individual muscle actions and inter-muscular coordination at the lower-limb joints could influence performance during linear and curvilinear movements in complex ways that were beyond the scope of the present study [6,65].

In addition, participants were recruited exclusively from the youth teams of professional clubs that had reached the final four of their competitive leagues. This selective recruitment of high-performing athletes may introduce a potential selection bias and may limit the generalizability of the findings to basketball players competing at lower or recreational levels. Future studies should consider recruiting players from a broader range of competition levels to strengthen the generalizability of the results.

Furthermore, although biological maturation, competition level, and training volume were accounted for through the inclusion and exclusion criteria, position-specific playing demands and individual differences in training history could not be controlled. These uncontrolled factors may have influenced the observed force-time metrics and should be considered when interpreting the findings of the study.

Finally, our sample consisted exclusively of young male basketball players, which limits the generalizability of the findings to female players or other age groups. Including female players and athletes of different ages in future research could help address these limitations and further strengthen the generalizability of the current findings.

## Conclusions

In conclusion, this study provides evidence that fast and slow SSC function and the rate of force development, but not maximal isometric strength, differ between fast and slow CS performers among young male basketball players. This study adds to the growing body of literature emphasizing the unique biomechanical demands and neuromuscular capabilities needed for multidirectional speed in basketball. Given the critical role of CS in basketball, developing and monitoring neuromuscular capacities, specifically rapid force production and absorption, in young athletes may contribute to long-term athletic success. Therefore, sports scientists and practitioners may consider incorporating training stimuli targeting fast and slow SSC function and rapid force expression to support CS performance in young basketball players, while acknowledging that randomized controlled trials are needed to further examine the effects of such training.
