# Correction: Assessing curvilinear sprint performance in basketball: The role of stretch-shortening cycle and isometric force-time metrics

**DOI:** 10.1371/journal.pone.0362123

**Published:** 2026-11-03

**Authors:** Caner Mavili, Ekrem Yilmaz, Huseyin Celik, Evrim Unver, Sukru Alpan Cinemre

S1 Appendix is omitted from the list of Supporting Information. It can be viewed below.

## Supporting information

S1 Appendix Definitions of force-time metrics. Names, abbreviations, units, and definitions of the force-time metrics derived from the drop jump (Appendix A), countermovement jump (Appendix B), and isometric mid-thigh pull (Appendix C) tests, with representative force-time curves illustrating the phases of each test.
(PDF)

## References

[1] Mavili C, Yilmaz E, Celik H, Unver E, Cinemre SA. Assessing curvilinear sprint performance in basketball: The role of stretch-shortening cycle and isometric force-time metrics. PLoS One. 2026;21(8):e0356775. doi: 10.1371/journal.pone.0356775 42647500 PMC13513976

