# Supplementary material for: Correction: Assessing curvilinear sprint performance in basketball: The role of stretch-shortening cycle and isometric force-time metrics
Source: PLoS One. 2026 Nov 3;21(11):e0362123. doi: 10.1371/journal.pone.0362123 (PMC13730332; doi:10.1371/journal.pone.0362123)
Supplement: Supplementary file 1 [file pone.0362123.s001.pdf]

Appendix A – Drop Jump (DJ) Metrics (Celik et al., 2024)

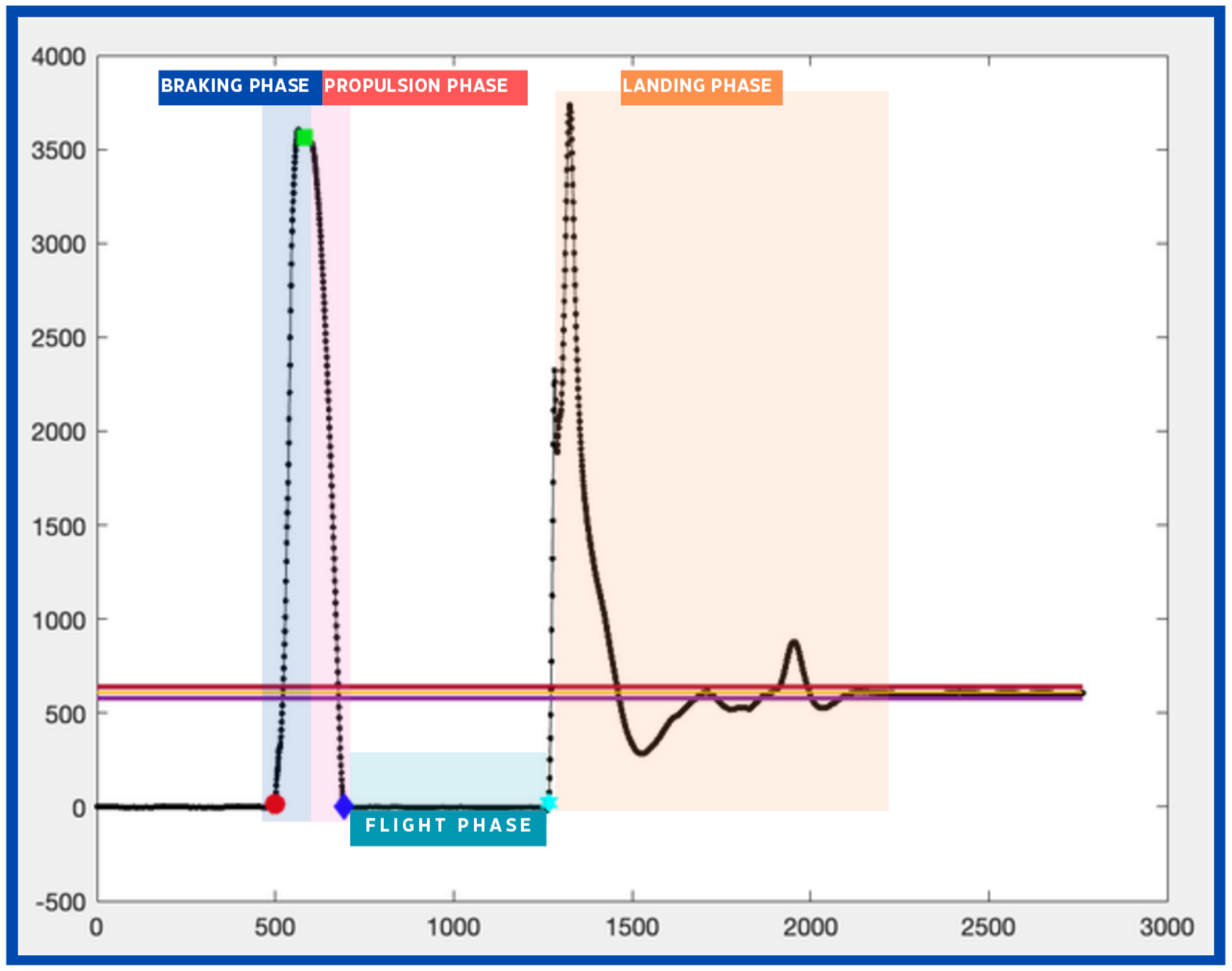

Measure no	Code measure name	Measure long name	Abbrv	Unit	Definition
1	JH	Jump Height	JH	meters	The peak displacement attained by the body center of mass (BCOM) during the flight phase calculated using the flight time in the air
2	GCT	Ground Contact Time	GCT	second(s)	The time spent on the ground after landing until takeoff
3	RSI	Reactive Strength Index	RSI	seconds/seconds	RSI was used to quantify SSC performance and was calculated as the ratio of JH to GCT
4	LPow	Average Power Eccentric Phase	LPow	W/kg	The mean mechanical power applied to the BCOM during the braking phase
5	TPow	Average Power Concentric Phase	TPow	W/kg	The mean mechanical power applied to the BCOM during the propulsion phase
6	PCOMD	Peak Center of Mass Displacement	PCOMD	meters	The maximum displacement value of the vertical displacement data points during the contact phase after the instant of landing onto the force plate.
7	pVGRF	Peak Vertical Ground Reaction Force	pVGRF	BW	PGRF calculated as the global maximum value of the VGRF data points in the contact phase (braking + propulsion)
8	LPF	Landing Peak Force	LPF	BW	The peak VGRF applied to the BCOM during the braking phase
9	TPF	Take-off Peak Force	TPF	BW	The peak VGRF applied to the BCOM during the propulsion phase
10	LTTD	Landing to Take-off Time Difference	LTTD	%	Braking phase peak force timing to propulsion phase peak force timing difference
11	Kvert	Vertical Stiffness	Kvert	N/m/kg	Kvert was calculated as the ratio of the peak VGRF during the contact phase of the jump to the displacement of the BCOM at the time of the peak VGRF. It was then normalized by the body mass.
12	BPT	Braking Phase Time	BPT	second(s)	The time taken to complete the braking phase
13	PPT	Propulsion Phase Time	PPT	second(s)	The time taken to complete the propulsion phase
14	Braking Impulse	Braking Impulse	Braking Impulse	m/s	The vertical impulse applied to the BCOM during the braking phase
15	Propulsion Impulse	Propulsion Impulse	Propulsion Impulse	m/s	The vertical impulse applied to the BCOM during the propulsion phase
16	SLC	Spring Like Correlation	SLC	unitless	A Pearson product-moment correlation between the VGRF applied to the BCOM and the vertical displacement of the BCOM in the contact phase.
17	SLB	Spring Like Behaviour	SLB	unitless	A classification that evaluates the function of the stretch-shortening cycle according to Pedley et al. (2022).

Appendix B – Counter Movement Jump (CMJ) Metrics (Linthorne, 2001; Comfort et al., 2018)

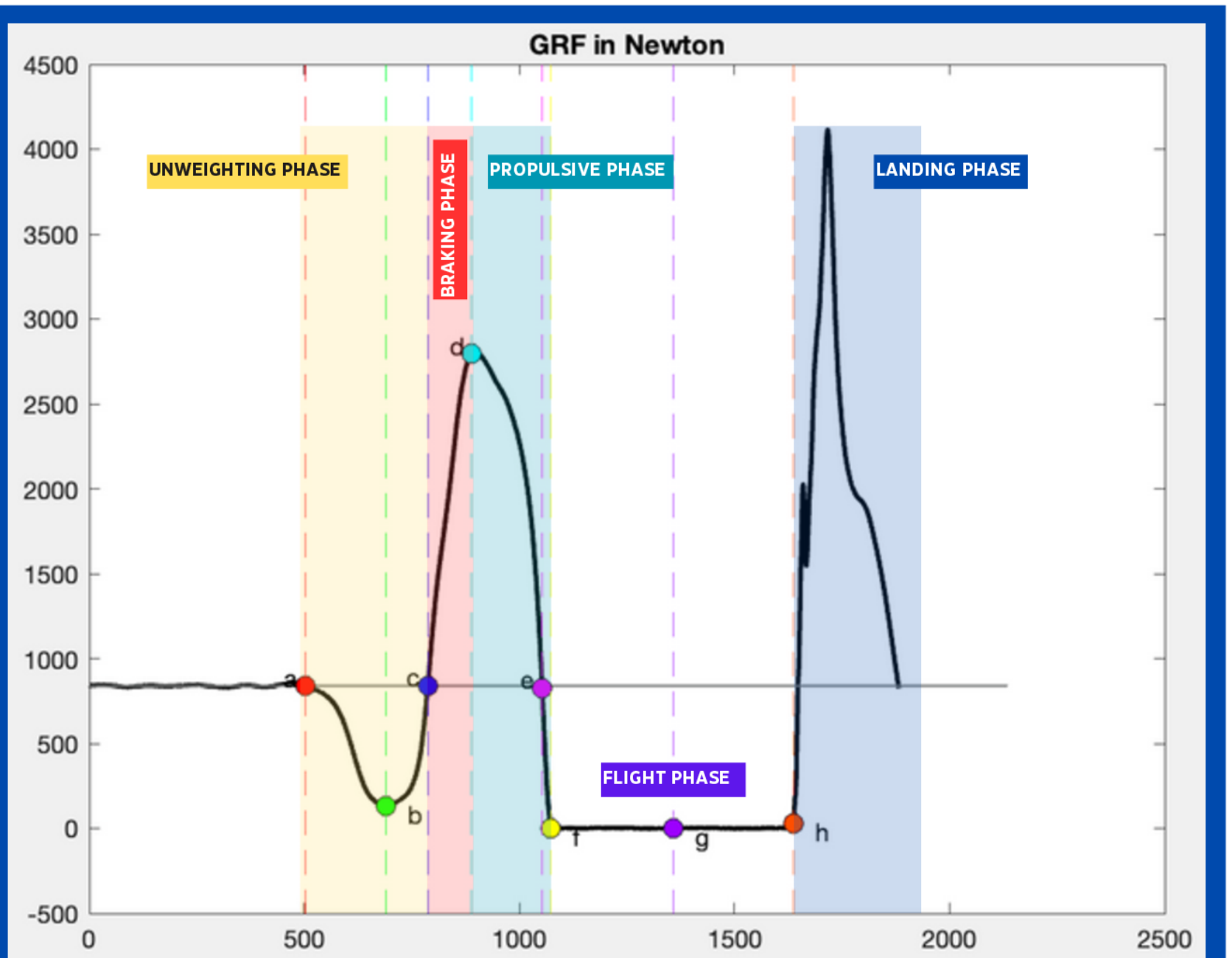

- a = Point a is start of the jump
- b = Point b represents the point at which the jumper's center of mass experiences the greatest downward acceleration.
- c = At the point C, the ground reaction force matches the body weight, resulting in a net force of zero on the jumper and no acceleration of the jumper's center of mass.
- d = Point d is the lowest point of the countermovement, where the jumper's center of mass is temporarily at rest (with zero velocity).
- e = The ground reaction force has decreased to match the body weight. As a result, the net force on the jumper and the acceleration of the jumper's center of mass are zero. Point e indicates the maximum upward velocity.
- f = Point g is the moment of takeoff, where the ground reaction force becomes zero for the first time.
- g = Point g marks the peak of the jump, where the jumper's center of mass is momentarily at rest.
- h = Point h is the moment of landing when the feet first contact the ground. The ground reaction force has a sharp "impact peak".

a -b = The jumper relaxes their leg and hip muscles, allowing the knees and hips to bend due to the effects of gravity.

b -c = The jumper's center of mass is still moving downward, but the jumper begins to increase leg muscle activation, reducing the downward speed.

c – d = The resultant force is now positive, and so the jumper is accelerating upwards, but still moving downwards.

d – e = This is the 'pushoff' phase, where the jumper moves upwards by extending the knees and hips. The velocity is now positive (upwards)..

e – f = The GRF drops below body weight, so the resultant force on the jumper and the acceleration of the jumper's center of mass are negative.

f – g = The only force acting on the jumper is his/her weight.

g – h = This is the descent phase of the flight, where the jumper's center of mass moves downward (with negative velocity) and the speed increases.

Measure no	Code measure name	Measure long name	Abbrv	Unit	Definition
1	TTT	Time To Take-off	TTT	second(s)	Total time from the initiation of movement to instant of take-off
2	FT	Flight Time	FT	second(s)	The time taken to complete the flight phase
3	JH	Jump Height	JH	meters	The peak displacement attained by the body center of mass (BCOM) during the flight phase calculated using the flight time in the air
4	RSI	Reactive Strength Index	RSI	seconds/seconds	The time taken to complete the flight phase divided by TTT
5	mRSI	Modified Reactive Strength Index	mRSI	m/s	The JH divided by the TTT
6	UPT	Unweighting Phase Time	UPT	second(s)	The time taken to complete the unweighting phase
7	BPT	Braking Phase Time	BPT	second(s)	The time taken to complete the braking phase
8	PPT	Propulsive Phase Time	PPT	second(s)	The time taken to complete the propulsion phase
9	MBF	Mean Braking Force	MBF	Body Weight (BW)	The mean vertical ground reaction force (VGRF) applied to the BCOM during the braking phase
10	MPF	Mean Propulsion Force	MBF	BW	The mean VGRF applied to the BCOM during the propulsion phase
11	MBP	Mean Braking Power	MBP	W/kg	The mean mechanical power applied to the BCOM during the braking phase
12	MPP	Mean Propulsion Power	MBP	W/kg	The mean mechanical power applied to the BCOM during the propulsion phase
13	MBV	Mean Braking Velocity	MBV	m/s	The mean vertical velocity of the BCOM during the braking phase
14	MPV	Mean Propulsion Velocity	MBV	m/s	The mean vertical velocity of the BCOM during the propulsion phase
15	PBF	Peak Braking Force	PBF	BW	The peak VGRF applied to the BCOM during the braking phase
16	PPF	Peak Propulsion Force	PPF	BW	The peak VGRF applied to the BCOM during the propulsion phase
17	PBP	Peak Braking Power	PBP	W/kg	The peak mechanical power applied to the BCOM during the braking phase

18	PPP	Peak Propulsion Power	PPP	W/kg	The peak mechanical power applied to the BCOM during the propulsion phase
19	PBV	Peak Braking Velocity	PBV	m/s	The peak vertical velocity of the BCOM during the braking phase
20	PPV	Peak Propulsion Velocity	PPV	m/s	The peak vertical velocity of the BCOM during the propulsion phase
21	netBrakingImpulse	Net Braking Impulse	NBI	m/s	The net vertical impulse applied to the BCOM during the braking phase
22	netPropulsionImpulse	Net Propulsion Impulse	NPI	m/s	The net vertical impulse applied to the BCOM during the propulsion phase
23	netImpulseRatio	Net Impulse Ratio	NIR	unitless	The ratio between the NPI to the NBI
24	brakingWork	Braking Phase Work	BPW	Joule	The integration of the net power applied to BCOM during the braking phase
25	propulsionWork	Propulsion Phase Work	PPW	Joule	The integration of the net power applied to BCOM during the propulsion phase
26	workRatio	Net Work Ratio	NWR	unitless	The ratio between the PPW to BPW
27	cmDepth	Countermovement Depth	CMD	meters	The peak negative vertical displacement of the BCOM
28	forceAtMinimum Displacement	Force at Minimum Displacement	FMD	BW	The VGRF applied to the BCOM at the point of the peak negative vertical displacement of the BCOM
29	jumpMomentum	Jump Momentum	JM	kg*m/s	The vertical momentum of the BCOM at the instant of take-off
30	braking_P_percent	Braking Phase Ratio	BPR	%	The time taken to complete the braking phase relative to the TTT
31	propulsion_P_percent	Propulsion Phase Ratio	PPR	%	The time taken to complete the propulsion phase relative to the TTT
32	unweighting_P_percent	Unweighting Phase Ratio	UPR	%	The time taken to complete the unweighting phase relative to the TTT
33	abs_Stiffness	Absolute Stiffness	AST	kN/m	FMD divided by the CMD
34	normalized_Stiffness	Normalized Stifness	NST	N/m/kg	AST normalized by body mass
35	unweighting_RFD	Unweighting Phase Rate of Force Development	URFD	BW/s	The average slope of the VGRF applied to the BCOM during the unweighting phase
36	eccentric_RFD	Eccentric Rate of Force Development	ERFD	BW/s	The average slope of the VGRF applied to the BCOM between point b and d
37	peakLandingForce	Peak Landing Force	PLF	BW	The peak VGRF applied to the BCOM during the landing phase

Appendix C – Isometric Mid-Thigh Pull (IMTP) Metrics (Guppy et al., 2022; Haff et al,. 2005)

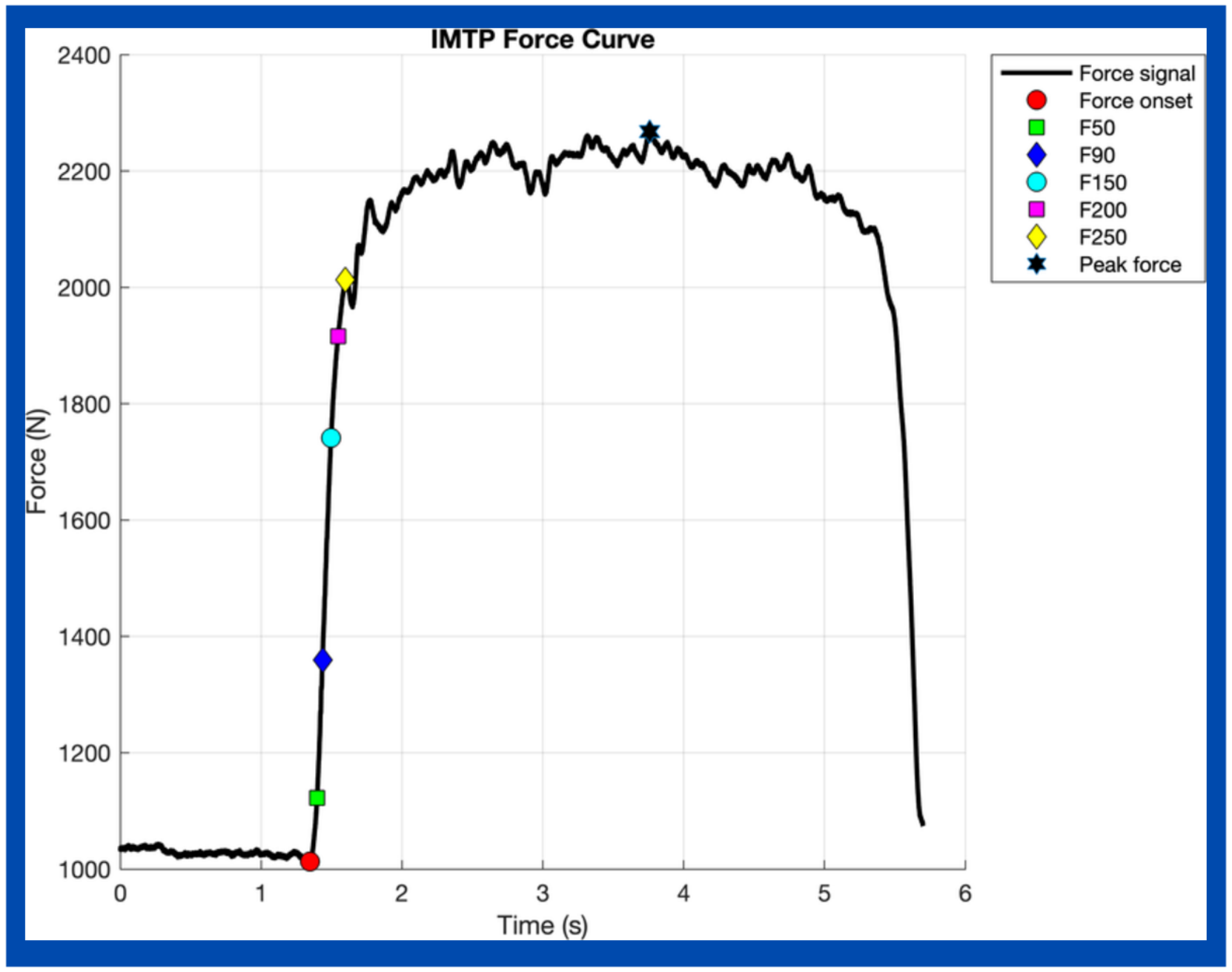

Measure no	Code measure name	Measure long name	Abbrv	Unit	Definition
1	Peak Force	Peak Force	Peak Force	Newton (N)	The peak VGRF applied during the test
2	Force 50 ms	Force at 50 millisecond (ms)	Force 50 ms	N	The instant VGRF at 50 ms from force-onset
3	Force 90 ms	Force at 90 ms	Force 90 ms	N	The instant VGRF at 90 ms from force-onset
4	Force 150 ms	Force at 150 ms	Force 150 ms	N	The instant VGRF at 150 ms from force-onset
5	Force 200 ms	Force at 200 ms	Force 200 ms	N	The instant VGRF at 200 ms from force-onset
6	Force 250 ms	Force at 250 ms	Force 250 ms	N	The instant VGRF at 250 ms from force-onset
7	Max pRFD20	Peak Rate of Force Development	Max pRFD20	N/s	The peak RFD with 20 ms sampling window, Haff et al. (2015)
8	RFD 50 ms	Rate of Force Development at 50 ms window	RFD 50 ms	N/s	The VGRF difference between 50 ms and force-onset divided by 50 ms
9	RFD 90 ms	Rate of Force Development at 90 ms window	RFD 90 ms	N/s	The VGRF difference between 90 ms and force-onset divided by 90 ms
10	RFD 150 ms	Rate of Force Development at 150 ms window	RFD 150 ms	N/s	The VGRF difference between 150 ms and force-onset divided by 150 ms
11	RFD 200 ms	Rate of Force Development at 200 ms window	RFD 200 ms	N/s	The VGRF difference between 200 ms and force-onset divided by 200 ms
12	RFD 250 ms	Rate of Force Development at 250 ms window	RFD 250 ms	N/s	The VGRF difference between 250 ms and force-onset divided by 250 ms
13	IMP 50 ms	Impulse at 50 ms window	IMP 50 ms	N.s	The vertical impulse between 50 ms and force-onset
14	IMP 90 ms	Impulse at 90 ms window	IMP 90 ms	N.s	The vertical impulse between 90 ms and force-onset
15	IMP 150 ms	Impulse at 150 ms window	IMP 150 ms	N.s	The vertical impulse between 150 ms and force-onset
16	IMP 200 ms	Impulse at 200 ms window	IMP 200 ms	N.s	The vertical impulse between 200 ms and force-onset
17	IMP 250 ms	Impulse at 250 ms window	IMP 250 ms	N.s	The vertical impulse between 250 ms and force-onset
